# Sleep hygiene and sleep control mediate the relationship between cognitive fusion and insomnia severity

**DOI:** 10.1111/bjc.70072

**Published:** 2026-05-27

**Authors:** Amanda L. Richdale, Francesco R. De Maria, Eric M. J. Morris

**Affiliations:** ^1^ Olga Tennison Autism Research Centre, School of Psychology and Public Health La Trobe University Melbourne Victoria Australia; ^2^ Department of Psychology, Therapy and Counselling, School of Psychology and Public Health La Trobe University Melbourne Victoria Australia

**Keywords:** cognitive fusion, insomnia, mediation analysis, sleep control, sleep hygiene

## Abstract

**Objectives:**

Insomnia is associated with mental ill health, worry and rumination—the two latter contribute to pre‐sleep cognitive arousal. These variables also associate with psychological inflexibility, including cognitive fusion (CF), a literal attachment to unhelpful thoughts, patterns and rules. Good sleepers generally have low CF, good sleep hygiene and a belief in sleep control, but individuals high in CF may have unhelpful beliefs about sleep hygiene and sleep control.

**Methods:**

We investigated associations between CF, sleep hygiene and sleep control, hypothesizing that each would contribute significant variance to insomnia and mediate the relationship between CF and insomnia.

**Results:**

Participants were 411 adults (*M*_*age*_ = 45.9 years, *SD* = 15.6) completing an online survey including demographics, and questionnaires about insomnia (ISI), CF (CFQ), sleep hygiene (SHS) and sleep control (BRISC). Hierarchical regression accounted for 34% of variance in insomnia (*p* < .001), showing that after controlling for demographic variables, CF, sleep hygiene and sleep control each accounted for significant variance in insomnia, with sleep control the strongest predictor (*β* = −.304). Parallel mediation modelling accounting for 22.2% of variance in insomnia (*p* < .001) showed both sleep hygiene and sleep control partially mediated the CF and insomnia relationship.

**Conclusions:**

Our study shows for the first time that CF, sleep hygiene and sleep control beliefs are inter‐related in their impact on insomnia. As CF is a core component of psychological inflexibility, ACT plus behaviour therapy (ACT‐I) may be a more suitable insomnia intervention than CBT plus behaviour therapy (CBT‐I) for some people.

**Practitioner points**

- Cognitive fusion, sleep hygiene and sleep control are key predictors of insomnia severity.
- Sleep hygiene and control partially mediate the link between cognitive fusion and insomnia.
- Higher cognitive fusion is associated with poorer sleep hygiene and weaker sleep control beliefs.
- Acceptance and Commitment Therapy for insomnia (ACT‐I) may offer advantages over traditional CBT‐I for some clients by directly targeting cognitive fusion in the treatment of insomnia.

## INTRODUCTION

About 30% to 45% of the adult population report insomnia which is associated with poor sleep quality, sleep onset and maintenance difficulties, short sleep and lack of refreshment on waking (Adams et al., 2017; Vargas et al., 2020) while world‐wide 12.4% of people meet criteria for insomnia disorder (van Straten et al., 2025). Insomnia is also associated with mental ill health, poor emotion regulation and fatigue. Factors that may contribute to insomnia include genetics, adverse childhood events, recent trauma or stress, increased cognitive activity and rumination, aging and female sex (Van Someren, 2021). The hyperarousal model of insomnia proposes that overactive neurobiological and psychological responses to environmental and psychological stressors create an over‐aroused state that is not conducive to sleep (Dressle & Riemann, 2023; Kalmbach et al., 2020). Physiological mechanisms that can contribute to hyperarousal include increased sensory sensitivity, increased core body temperature and HPA axis activity (e.g. cortisol) during sleep, and changes in specific aspects of the sleep EEG. Cognitive arousal is associated with increased pre‐sleep rumination and worry, emotional distress and related dysfunctional beliefs about sleep (Dressle & Riemann, 2023).

Insomnia and mental ill health are also associated with psychological inflexibility (PI) (Castro et al., 2021; McCracken et al., 2020; Peltz et al., 2020), which is a multi‐dimensional pattern of psychological rigidity in which people's decisions and behaviours are excessively governed by internal experiences (thoughts, emotions, urges) at the expense of actions guided by personally held values (Bond et al., 2011; Hayes, 2004). In both general population and clinical samples, increased PI can promote disturbed sleep (Ryan et al., 2025). One of the six psychological processes contributing to PI is cognitive fusion (CF), which is associated closely with worry, anxiety, rumination and an over‐active mind (Miniati et al., 2023; Zakiei et al., 2024). Those with high CF tend to have a literal attachment to unhelpful thoughts, thought patterns and rules that prevent external engagement with life in line with values (Gillanders et al., 2014; Hayes et al., 2006), thus, they are at increased risk of insomnia and mental ill health (Daly‐Eichenhardt et al., 2016; Peltz et al., 2020; Zakiei et al., 2024; Zhang et al., 2021).

The association of increased CF with rumination, worry and anxiety may make pre‐sleep de‐arousal processes more difficult. A study (*N* = 323) by Zhang et al. (2021) reported that CF acted as a mediator between occupational stressors and poor sleep; in a clinical sample of adults with pain (*N* = 252) improvement in insomnia was associated with decreased CF (Daly‐Eichenhardt et al., 2016); and CF was the strongest PI predictor of insomnia in a non‐clinical sample of adults with pain (*N* = 1657) (McCracken et al., 2023). Examination of the effect of five PI processes (experiential avoidance, CF, lack of present moment awareness, limited perspective taking and inaction) on anxiety, pre‐sleep arousal and sleep quality in a community sample of adults (*N* = 704) showed that while all PI processes were associated with reduced sleep quality, CF showed the strongest relationship (Mastin‐Purcell et al., 2025). Modelling the effect of these five processes on sleep quality mediated by pre‐sleep arousal and anxiety, CF—through both somatic and cognitive pre‐sleep arousal—had the strongest effect on sleep quality, with the effect of CF on sleep through cognitive arousal being strongest (Mastin‐Purcell et al., 2025), supporting that CF is an important factor in the maintenance of insomnia, most probably via increased pre‐sleep cognitive arousal.

Increased cognitive and somatic pre‐sleep arousal is also associated with high sleep control need (Brosschot et al., 2010; Gilbert et al., 2019), and young adults who believe sleep is not under their control have higher PI and increased sleep disturbance (Peltz, 2024). Many people also ruminate over how little they may sleep (Lancee et al., 2017), with associated stress about sleep then heightening stress‐related physiological markers and creating hyper‐vigilance, preventing sleep (Marques et al., 2016). Conversely, belief that one has control over sleep timing, length and quality is related to better sleep, while anxiety about lack of sleep control and low self‐efficacy concerning sleep onset and maintenance impacts insomnia severity for many adults (Grandner et al., 2020). Therefore, individuals with a high need for sleep control or low sleep control beliefs may be expected to have increased CF.

Sleep hygiene, while often poorly defined, involves the promotion of habits conducive to good sleep and is recommended for good sleep either on its own, or as a component of insomnia intervention (i.e. CBT‐I; De Pasquale et al., 2024). It is associated with small to medium (Chung et al., 2018; Friedrich & Schlarb, 2018) effect size improvements in insomnia symptoms However both subjective and objective improvement in insomnia symptoms is inferior to other non‐pharmacological interventions including CBT‐I (Ruan et al., 2025). Sleep hygiene teaches sleep preparedness and external conditions conducive to sleep (De Pasquale et al., 2024; Ruan et al., 2025) via relaxing activities prior to bed, and regular bedtimes and wake times to promote de‐arousal prior to sleep. Other recommendations concern the sleep environment (e.g. cool room temperature, low noise and light and no screen access) and daily routines (e.g. timing of meals, exercise and light exposure) that can help align the sleep–wake cycle with desired sleep and wake times. Poor sleep hygiene has been associated with insomnia severity and mental ill health (Alanazi et al., 2023; Galvin et al., 2022), acting as both a partial mediator and a moderator of the relationship between anxiety and insomnia in nurses (Chou et al., 2015), while in university students poor sleep hygiene was associated with increased anxiety (Manzar et al., 2021). Consequently, poor sleep hygiene can affect both sleep and mental health, most likely by impeding activities that promote de‐arousal prior to sleep and can be prominent in the maintenance of insomnia. The rumination, anxiety and attachment to unhelpful rules that are associated with high CF may also interfere with an individual's ability to implement good sleep hygiene habits.

Therefore, CF negatively affects sleep via increased pre‐sleep cognitive arousal, but it may also have a negative effect on beliefs and rules about sleep control and sleep hygiene. Consequently, some of the negative impact of CF on sleep may occur via one or both of sleep control and sleep hygiene. Hence, the aim of this study was to identify the relationships between CF, sleep control, sleep hygiene and insomnia symptom severity. We hypothesized that (1) CF, sleep hygiene and sleep control would be significantly associated with insomnia symptom severity; (2) that sleep hygiene, sleep control and CF would each account for significant variance in insomnia symptom severity; and (3) using a parallel mediation model, both sleep hygiene and sleep control would mediate the relationship between cognitive fusion and insomnia symptom severity.

## METHOD

### Participants

Participants were a convenience sample of 411 Australian and New Zealand residents aged from 18 to 69 years who completed an anonymized, online survey about psychological flexibility, mental health, wellbeing and sleep.

### Measures

Demographics variables included participants' age, sex at birth, gender identity, country of birth, Indigenous identity, cultural background/ethnicity^1^ (based on the Australian Bureau of Statistics [ABS] census), language spoken at home, highest level of education, employment, physical health diagnoses, mental health diagnoses, diagnosed sleep disorders (e.g. *Have you been diagnosed with any of the following sleep disorders?*; an ‘*other*’ option was also available), and non‐sleep and sleep medications.

#### Cognitive fusion (CFQ; Gillanders et al., 2014)

The CFQ measures the tendency for a person's behaviour to be overly regulated and influenced by cognition. It has good internal consistency (*α* = .90) in student and community samples (Gillanders et al., 2014) and excellent internal consistency in the current sample (*α* = .95). The CFQ consists of 7 Likert‐scale items scored between 1, ‘never true’ and 7, ‘always true’, with higher total scores indicating increased cognitive fusion. The mean score reported across a student and community sample was 22.28, *SD =* 8.30 (Gillanders et al., 2014).

#### Sleep hygiene index (SHI; Mastin et al., 2006)

This is a 13‐item measure of sleep hygiene with adequate reported internal consistency (*α* = .66) and acceptable internal consistency in the current sample (*α* = .77). Likert‐scale items ask the frequency of participants' behaviours, and are scored between 0, ‘Never’ and 4, ‘Always’. Higher total scores indicate poorer sleep hygiene behaviours with a mean score of 34.66, *SD* = 6.6 across a sample of university students (Mastin et al., 2006).

#### Brief index of sleep control (BRISC; Grandner et al., 2020)

This is a brief 4‐item measure of how much participants believe they have control over their sleep. It had good internal consistency (*α* = .80) in a community sample of 22–60‐year‐olds (Grandner et al., 2020) and in the current sample (*α* = .80). Likert‐scale items ask participants how much they feel they have control over four aspects of their sleep behaviour with ratings between 0, ‘None at all’ and 4, ‘Complete control’. Higher mean scores indicate better perceived control over sleep behaviour, with a mean score of 1.93, *SD* = 92 reported for a community sample of adults (Grandner et al., 2020).

#### Insomnia severity index (ISI; Bastien et al., 2001)

This is a 7‐item measure of the severity of insomnia symptoms and has good internal consistency with *α* = .90 and .91 in community and clinical samples respectively (Morin et al., 2011) and *α* = .89 in the current sample. Item scores range from 0 to 4, with higher scores indicating increased difficulties. Total scores <8 indicate no insomnia, scores ≥10 show the best specificity and sensitivity for insomnia in a community sample and scores ≥15 indicate moderate to severe insomnia (Morin et al., 2011).

### Procedure

The La Trobe University Human Ethics Committee (HEC22121) approved the study (please see National Statement on Ethical Conduct in Human Research 2023 | NHMRC). Data for the study were drawn from a larger survey designed to examine psychological flexibility, mental health, sleep and wellbeing, conducted from 14 July to 24 August, 2022. Prior to going live, the survey was tested by the research team for any errors. Participants were recruited through paid Facebook advertising targeting Australian and New Zealand residents, two La Trobe University participant registries and snowball sampling via the study team's personal Facebook pages. The study was hosted online through REDCap (Harris et al., 2009; Harris et al., 2019).

The online participant information statement explained the study including that English proficiency was needed, and those who were interested provided informed consent. Inclusion criteria were (1) aged 18–69 years and (2) a current resident of Australia or New Zealand and those who did not meet both inclusion criteria prior to the survey entry exited at that point. The survey took about 30 min to complete. A bot check was used when participants first entered the survey site and two attention check items in the survey asked participants to select a specific response. As a thank‐you for participation on survey completion, participants could enter a draw for one of 10 gift cards worth $50 AUD for Australian residents and $40 USD for New Zealand residents; participant details could not be linked to their survey data.

### Data analysis

An a priori power analysis for linear multiple regression (fixed model, *R* ^2^ deviation from zero) was conducted using G*Power 3.1.9.7 (Faul et al., 2007, 2009) to determine the minimum sample size required. With three predictors, an effect size of *f* ^2^ = .07, power of .80 and *α* of .05, the analysis indicated a minimum required sample size of *N* = 160.

Data were analysed using the IBM Statistical Package for the Social Sciences (SPSS; version 29). Participants who successfully completed only one of the two attention checks were reviewed for inconsistencies and FD, and two members from the larger study decided whether they were retained. Those with missing data, completion times of 10 min or less or who failed both attention checks (*n* = 79) were removed from the dataset. Those who did not complete all survey questionnaires relevant to this study (*n* = 268) were also excluded, leaving 411 participants with complete data for the current analyses.

Descriptive statistics were calculated for all variables; dichotomous variables were coded 0 (no/not present) or 1 (yes/present); sex was coded 1 (male) or 2 (female)—no‐one identified as intersex at birth. As the ABS cultural/ethnicity categories do not include ‘New Zealand’, participants in the ‘Other’ who identified as New Zealander or pakeha (Māori for white person/European) were reclassified as ‘New Zealander’. Reported ‘Other’ categories for physical and mental health conditions were checked and reclassified as needed; heart disease, blood pressure and stroke were classified as ‘cardiovascular condition’. Three new categories were created from physical health ‘other’ responses: Chronic Fatigue Syndrome, Fibromyalgia and Arthritis. Within mental health conditions, attention deficit disorder and ADHD were classified as Attention Deficit Hyperactivity Disorder, and the range of terminologies entered for an autism diagnosis were all classified as Autism Spectrum Disorder. Similar checks were completed for sleep disorders and medications. Participants who reported taking melatonin or medication for sleep, but did not report a sleep disorder, were classified as having a ‘sleep problem’.

Normality, outliers and multicollinearity were checked. Where data were not normal, non‐parametric or bootstrap analyses were conducted. Spearman's correlations were calculated for questionnaire total scores, age, sex at birth and number of reported mental and physical health conditions. All correlations were <.70 thus multicollinearity was not a problem. A 1000 bootstrapped hierarchical regression predicting insomnia symptom severity (ISI total score) was then conducted. We planned to enter age and sex at Step 1; number of physical and mental health conditions at Step 2; CF (CFQ total score) at Step 3; and both sleep control (BRISC) and sleep hygiene (SHI) scores at Step 4 to determine if they added significant additional variance after the contribution of CF was accounted for. A parallel mediation model was planned (Hayes' PROCESS Model 4 with 5000 bootstrap samples: Hayes, 2018) with sleep control (BRISC) and sleep hygiene (SHI) explored as mediators of the CI (CFQ)—insomnia (ISI) relationship.

## RESULTS

### Sample demographics and health

Participants' average age was 45.9 years (*SD* = 15.6, *N* = 411) and most were female at birth (80%); females at birth were significantly younger than males at birth (U = 10805.0, *z* = −2.79, *p* = .005). Within the sample, 75.6% currently identified as female and 19% identified as male, with the remainder selecting an LBGTQI option, including 3.9% who selected more than one gender and two who preferred not to say. Most participants were born in Australia, identified as Australian, spoke English at home and had a post‐secondary school education (Table 1).

**TABLE 1:** Demographic descriptions of study participants.

Characteristics	
*Sex* ^a^	*Age*
Female at birth (*n* = 329)	44.8 years (*SD* = 15.4)
Male at birth (*n* = 82)	50.2 years (*SD* = 15.7)
*Country of birth* (*N* = 411)	*Percentage of participants* ^b^
Australia	69.6
New Zealand	11.2
Other	19.2
*Cultural identity/ethnicity* ^b^ (*N* = 411)	
Australian	60.8
Aboriginal and Torres Strait Islander^c^	1.2
New Zealander	5.8
Māori^d^	1.7
English	22.9
Irish	10.9
Scottish	10.9
Chinese	3.6
Italian	2.9
German	3.6
Indian	1.9
Greek	1.5
Vietnamese	1.2
Other	14.4
None	4.4
Prefer not to say	1.2
*Language spoken at home* (*N* = 411)	
English	97.3
Other	2.7
*Education* (*N* = 411)	
Not completed high school	4.9
Completed high school	10.7
Trade, certificate or diploma	23.0
Bachelor's degree	36.3
Master's or PhD	21.9
Other	2.7
*Employment* (*n* = 405)	
Full‐time	32.6
Part‐time/casual	29.6
Unemployed	9.6
Student/in training	10.6
Retired	13.3
Other	4.2

^a^ 95% of the sample identified with their sex at birth.

^b^ Percentages add to >100 as participants could select multiple backgrounds.

^c^ Aboriginal and Torres Strait Islander are Australian first nations peoples.

^d^ Māori are New Zealand first nations people.

Health conditions are reported in Table 2. Half the participants (53.3%) reported at least one physical health diagnosis, the most common being cardiovascular disease and obesity; more than half (62%) reported at least one mental health diagnosis, the most common being anxiety and depression. One or more sleep disorders or a sleep problem were reported by 30.9% of participants, the most common diagnosis being insomnia. Two thirds (66.4%) of participants took medication, and 12.7% reported that one of their medications was for sleep. Mean scores for the questionnaires were: CFQ (*M* = 30.06, *SD* = 10.01), SHI (*M* = 20.65, *SD* = 7.20), BRISC (*M* = 2.01, *SD* = .90) and ISI (*M* = 10.56, *SD* = 6.47). Using the clinical cutoff suggested for a community sample (≥10), about half the participants (53.8%) had an ISI score indicative of insomnia (Morin et al., 2011), with 27.4% having moderate–severe insomnia symptoms (ISI ≥15) suggesting clinical insomnia.

**TABLE 2:** Participant‐reported physical and mental health, and sleep diagnoses (*N* = 411).

Physical health condition^a^	%	Mental health condition^b^	%	Sleep disorder	%
None	47.4	None	38.2	Restless legs	7.3
Cardiovascular	17.0	Anxiety	46.0	Sleep apnoea	9.2
Diabetes	7.5	Depression	44.0	Insomnia	15.1
Obesity	14.6	Schizophrenia	1.2	Parasomnia	1.2
Asthma	12.9	Post‐traumatic stress disorder	18.5	Other	1.5
Allergy	8.0	Eating disorder	5.4	Sleep problem^c^	3.9
Epilepsy	1.5	Borderline personality disorder	4.1		
Hearing impairment	4.6	Bipolar disorder	2.4		
Visual impairment	1.7	Attention deficit hyperactivity disorder	10.9		
Arthritis	2.2	Autism spectrum disorder	6.8		
Fibromyalgia	2.7	Other	2.2		
Chronic fatigue	2.4				
Other	13.4				

^a^ 23.6% of participants had two or more physical health conditions.

^b^ 44.0% of participants had two or more mental health conditions.

^c^ Participants who reported taking medication for sleep, but had no diagnosed sleep disorder.

### Spearman's correlation

Correlations varied from negligible to strong (Table 3). Older age was weakly associated with more physical health problems, while being younger was weakly associated with more mental health problems and moderately associated with higher CF and poorer sleep hygiene. Any associations between sex or physical health and other variables were weak or negligible (all *r* < .20), though some of these associations were significant. Mental health conditions, CF and sleep hygiene were all significantly and moderately to strongly associated, but the correlations for sleep control were significant but weak. The strongest association was between CF and mental health conditions where more mental health conditions were associated with increased CF. Insomnia symptoms were most strongly associated with increased CF and lower sleep control.

**TABLE 3:** Spearman's correlations for age, sex, health, inflexibility and sleep variables (*N* = 411).

	Age	Sex	PH	MH	CFQ	SHI	BRISC
Sex	−.14						
	.005						
PH	.**25**	−.03					
	**<.001**	.599					
MH	**−.26**	.13	.16				
	**<.001**	.009	.002				
CFQ	**−.30**	.07	−.01	.**50**			
	**<.001**	.184	.902	**<.001**			
SHI	**−.39**	.05	.06	.**32**	.**46**		
	**<.001**	.266	.259	**<.001**	**<.001**		
BRISC	.07	.01	−.13	**−.26**	**−.29**	**−.16**	
	.19	.824	.007	**<.001**	**<.001**	**<.001**	
ISI	−.09	.05	.11	.**35**	.**44**	.**36**	**−.44**
	.06	.307	.032	**<.001**	**<.001**	**<.001**	**<.001**

*Note*: Bold values indicate all the significance values.

Abbreviations: BRISC, Brief Index of Sleep Control; CFQ, Cognitive Fusion Questionnaire; ISI, Insomnia Severity Index; MH, mental health condition; PH, physical health condition; SHI, Sleep Hygiene Index.

### Hierarchical regression with insomnia symptoms as the dependent variable

A hierarchical regression was used to determine if after accounting for age, mental and physical health conditions, CF (CFQ), sleep hygiene (SHI) and sleep control (BRISC) would account for a significant amount of variance in insomnia symptom severity (ISI); sex was not included as it only had a significant weak association with mental health conditions. Two participants had standardized residuals >3 (3.11; 3.03) but they were retained. The final model was significant and accounted for 34.4% of the variance in ISI scores, *R* ^*2*^ = .344, *F* (6, 404) = 35.34, *p* < .001. At the first step, age accounted for a non‐significant .6% of the variance in ISI scores, *R* ^*2*^ = .006 *F* (1, 409) = 2.62, *p* = .106. At step 2 number of mental and physical health problems together accounted for an additional 11.3% of variance, Δ*R* ^*2*^ = .113 Δ*F* (2, 407) = 25.98, *p* < .001. At step 3 the addition of the CFQ added a further 10.4% of variance, Δ*R* ^*2*^ = .113 Δ*F* (1, 406) = 54.20, *p* < .001. At step 4, the SHI and the BRISC were added, accounting for another 12.2% of variance Δ*R* ^*2*^ = .113 Δ*F* (2, 404) = 37.43, *p* < .001. All variables except physical health conditions were significant in the final model: Age (*β* = .127; 95% BCa CI [.014; .093], *p* = .007), number of mental health conditions (*β* = .121; 95% BCa CI [.128; 1.00], *p* = .016), CFQ (*β* = .218; 95% BCa CI [.077; .213], *p* < .001), SHI (*β* = .230; 95% BCa CI [.121; .293], *p* < .001) and BRISC (*β* = −.304; 95% BCa CI [−2.93; −1.53], *p* < .001). Beta values (non‐bootstrapped) showed that the BRISC made the strongest contribution to ISI scores in the final model.

### Parallel mediation model

As age and mental health conditions were significant in the final regression model, we initially ran our full mediation model with these two variables as covariates. Mental health conditions was significant (95% CI [.326; 1.134]), but age was not (95% CI [−.004; .072]). The full parallel mediation model with mental health conditions as a covariate was significant and accounted for 21.2% of the variance in insomnia symptoms, *R* ^*2*^ = .212, *F* (2, 408) = 54.822, *p* < .001. The total effect (95% CI [.170; .295]), direct effect (95% CI [.068; .197]), indirect effect (95% CI [.065; .137]) and the covariate (95% CI [.288; 1.093]) were all significant. The paths from CFQ to SHI (95% CI [.237; .373]), SHI to ISI (95% CI [.090; .255]), CFQ to BRISC (95% CI [−.122; −.048]) and BRISC to ISI (95% CI [−.712; −.408]) were also all significant. The model is presented in Figure 1 and shows that both sleep hygiene and sleep control act as partial mediators of the relationship between CF and insomnia symptoms. Cognitive fusion demonstrated a significant direct association with insomnia symptoms as well as significant indirect effects via both poor sleep hygiene and low sleep control.

**FIGURE 1:**
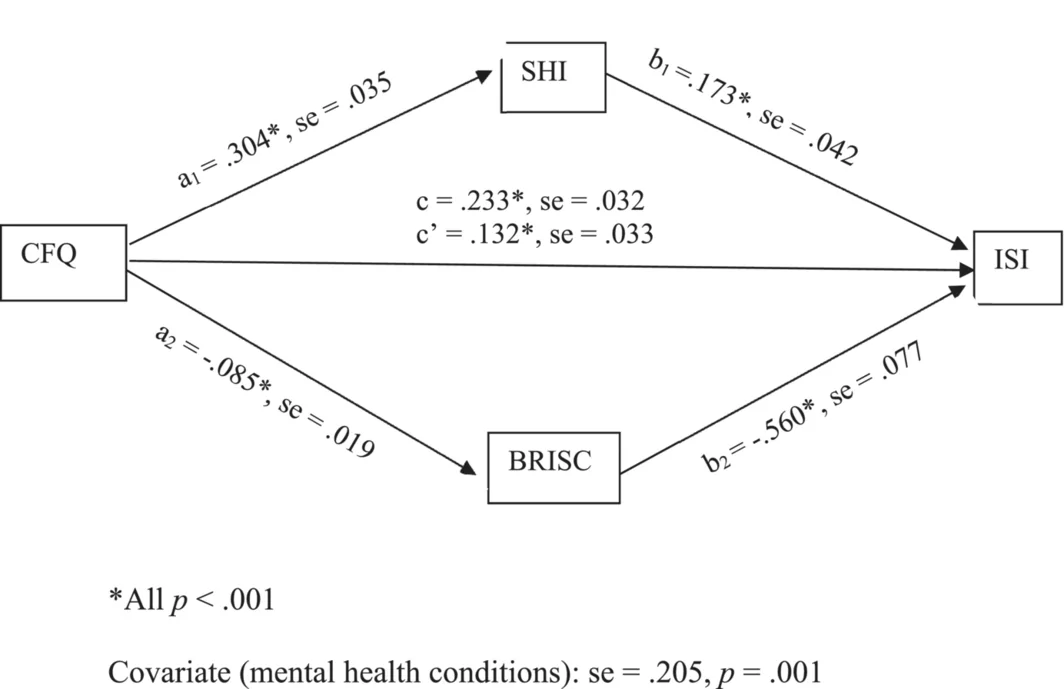
Parallel mediation model with sleep hygiene (SHI) and sleep control (BRISC) as mediators of the relationship between cognitive fusion (CFQ) and insomnia (ISI) and mental health conditions as a covariate.

## DISCUSSION

We examined the relationships between cognitive fusion (CF) using the CFQ, sleep control (BRISC), sleep hygiene (SHI) and insomnia (ISI) in a community sample of adults. About half our sample reported symptoms suggesting insomnia, with more than a quarter of these participants having symptoms consistent with clinical insomnia (Morin et al., 2011), tended to have increased cognitive fusion, reduced sleep hygiene habits and normal sleep control beliefs compared with the populations on which these questionnaires were standardized (Gillanders et al., 2014; Grandner et al., 2020; Mastin et al., 2006). We found that, as hypothesized, increased CF was significantly and moderately correlated with increased insomnia symptom severity and poorer sleep hygiene, and significantly but weakly associated with decreased sleep control. Similarly, after controlling for age and physical and mental health conditions, all three variables (CFQ, SHI, BRISC) contributed significant variance to insomnia symptom severity, with sleep control being the strongest predictor; age and number of mental health conditions also contributed significant variance to insomnia symptom severity. Overall, our model accounted for a third of the variance in insomnia symptom severity. Results supported the final hypothesis that sleep control and/or sleep hygiene would mediate the relationship between CF and insomnia symptoms, showing for the first time that both variables are partial mediators of the CF‐insomnia relationship. The correlation and regression outcomes are consistent with previous research showing that CF (Mastin‐Purcell et al., 2025; McCracken et al., 2023), sleep hygiene (Chou et al., 2015; Galvin et al., 2022), sleep control (Grandner et al., 2020) and mental health (Palagini et al., 2022) are all associated with insomnia symptoms.

Our results are consistent with recent research showing that components of psychological inflexibility (PI), including CF can negatively impact sleep (Ryan et al., 2025; Zakiei et al., 2024), with the effect of CF most likely being due to its impact on cognitive arousal (Mastin‐Purcell et al., 2025; Zakiei et al., 2024). While the present findings do not test intervention effects, they are consistent with the process model of Acceptance and Commitment Therapy (ACT), which addresses PI processes, and therefore provide a rationale for investigating ACT adapted to address insomnia (i.e. ACT‐I) in future clinical trials and studies examining its underlying change processes (Paulos‐Guarnieri et al., 2022; Ruan et al., 2022). For example, both ACT‐I and CBT‐I reduced insomnia, depression and fatigue in a sample of 30 sleep‐clinic patients (15 participants randomized to each condition), with ACT additionally reducing anxiety (Shin et al., 2023) and ACT‐I improved sleep quality in 10 patients who were CBT‐I non‐ or partial‐responders (Hertenstein et al., 2014). Cognitive fusion may be a key component of PI to address in individuals with insomnia given its stronger relationship with pre‐sleep cognitive arousal (Mastin‐Purcell et al., 2025), one of the primary components of the hyperarousal theory of insomnia (Dressle & Riemann, 2023).

We showed for the first time that sleep hygiene partially mediates the relationship between CF and insomnia symptoms. Sleep hygiene is a modifiable behaviour that may contribute to better sleep (Ruan et al., 2025). Psychological and behavioural sleep interventions generally address aspects of sleep hygiene, and our results indicate it is an important factor to include in any sleep intervention. However, which components of sleep hygiene are most important for good sleep or how it should be defined remain to be determined (De Pasquale et al., 2024). Additionally, the SHI (Mastin et al., 2006) used here includes two questions related to worry and negative emotions which may partially account for sleep hygiene's significant association with CF and insomnia symptoms. Clearly, further work is needed to determine when and how the various components thought to make up good sleep hygiene can lead to better sleep.

Feeling that sleep is not under one's control is associated with sleep disturbance and is related to sleep self‐efficacy; both are associated with beliefs about sleep control (Grandner et al., 2020; Peltz, 2024). According to Grandner et al. (2020) sleep control also encompasses external control, the lack of which may be due to familial and societal demands. We hypothesized that CF could lead to worry and anxiety about sleep control and thus be related to reduced sleep control, and it was. Our regression analysis also showed for the first time that sleep control was a stronger predictor of insomnia symptom severity than either CF or sleep hygiene and that it acted as a partial mediator of the CF‐insomnia relationship. This supports that modifiable sleep control beliefs should be a target of sleep intervention, which may be addressed by using ACT‐I or CBT‐I intervention for insomnia. The ability to deal with uncontrollable external demands that impact sleep control may best be addressed within ACT via mindfulness and values clarification, and with sleep education presented with flexibility (reducing the risk of rigid rules about sleep being reinforced) that assists the individual to plan and maximize their opportunities for sleep within their current external constraints. Additionally, CF—where thoughts are taken literally and dominate behaviour—is a form of psychological rigidity, impacting adaptiveness and learning from experience. Targeting CF in ACT supports increasing flexibility (Macri & Rogge, 2024), which may be particularly important in assisting people to try new behaviours such as sleep hygiene and accurate appraisals about sleep control and to orient to their effects.

### Strengths and limitations

A strength of our study was a well‐described, non‐clinical sample of adults with adequate sample size and sufficient power to investigate our questions of interest using valid and reliable questionnaires. Nevertheless, the study was cross‐sectional, preventing any causal inferences, self‐report and online, however we undertook a range of steps to ensure we had valid respondents. Additionally, 80% of our sample was female, over half reported a physical or mental health condition, and there were insufficient participants identifying as LBGTQI to examine gender; thus, the sample was not representative of the general population. Increased female participation is common in online research (Wu et al., 2022), and sex and physical health conditions were only very weakly (*r*_*s*_ < .20) associated with other variables, while the associations with mental health conditions are consistent with existing literature. Hence, we can have some confidence that our findings are more widely applicable.

## CONCLUSIONS

This study examined the relationships of cognitive fusion, sleep control and sleep hygiene with each other and with insomnia symptom severity for the first time. While each of these variables has been shown previously to individually impact insomnia (Chung et al., 2018; Grandner et al., 2020; Zakiei et al., 2024), our results showed that sleep control had a stronger effect on insomnia symptoms than either CF or sleep hygiene, with both sleep control and sleep hygiene partially mediating the CF‐insomnia relationship. Our findings suggest that relationships between these variables should be investigated longitudinally. Sleep control and sleep hygiene are important components of sleep interventions, and given their relationships with CF and thus psychological inflexibility, ACT‐I may be a more suitable insomnia intervention than CBT‐I for some people. Future research is needed to understand, both cross‐sectionally and longitudinally, the necessary and sufficient components of sleep hygiene and factors that may influence beliefs about sleep control. More research is also needed to determine the necessary and sufficient components of ACT‐I for clients with insomnia (e.g. see Looman et al., 2024).

## AUTHOR CONTRIBUTIONS

**Amanda L. Richdale:** Conceptualization; methodology; supervision; investigation; formal analysis; writing – original draft; writing – review and editing. **Francesco R. De Maria:** Conceptualization; methodology; investigation; writing – original draft; validation. **Eric M. J. Morris:** Conceptualization; methodology; supervision; investigation; formal analysis; project administration; writing – review and editing; data curation.

## FUNDING INFORMATION

This research was not supported by any granting body.

## CONFLICT OF INTEREST STATEMENT

The authors have no conflict of interest to report.

## ETHICS STATEMENT

The research was approved by the La Trobe University Human Ethics Committee (HEC22121).

## PARTICIPANT CONSENT

Participants provided informed consent after reading the online participant information statement.

## Data Availability

Please contact the corresponding author, Associate Professor Eric Morris.

## Footnotes

^1^
The Australian Bureau of Statistics does not collect data on race.

## References

[bjc70072-bib-0001] Adams, R. J. , Appleton, S. L. , Taylor, A. W. , Gill, T. K. , Lang, C. , McEvoy, R. D. , & Antic, N. A. (2017). Sleep health of Australian adults in 2016: Results of the 2016 Sleep Health Foundation national survey. Sleep Health, 3(1), 35–42. 10.1016/j.sleh.2016.11.005 28346149

[bjc70072-bib-0002] Alanazi, E. M. , Alanazi, A. M. M. , Albuhairy, A. H. , & Alanazi, A. A. A. (2023). Sleep hygiene practices and its impact on mental health and functional performance among adults in Tabuk City: A cross‐sectional study. Cureus, 15, e36221. 10.7759/cureus.36221 PMC10105495 37069886

[bjc70072-bib-0003] Bastien, C. H. , Valliéres, A. , & Morin, C. M. (2001). Validation of the Insomnia Severity Index as an outcome measure for insomnia research. Sleep Medicine, 2(4), 297–307. 10.1016/S1389-9457(00)00065-4 11438246

[bjc70072-bib-0004] Bond, F. W. , Hayes, S. C. , Baer, R. A. , Carpenter, K. M. , Guenole, N. , Orcutt, H. K. , Waltz, T. , & Zettle, R. D. (2011). Preliminary psychometric properties of the acceptance and action questionnaire–II: A revised measure of psychological inflexibility and experiential avoidance. Behavior Therapy, 42(4), 676–688. 10.1016/j.beth.2011.03.007 22035996

[bjc70072-bib-0005] Brosschot, J. F. , Verkuil, B. , & Thayer, J. F. (2010). Conscious and unconscious perseverative cognition: Is a large part of prolonged physiological activity due to unconscious stress? Journal of Psychosomatic Research, 69(4), 407–416. 10.1016/j.jpsychores.2010.02.002 20846542

[bjc70072-bib-0006] Castro, J. , Pereira, J. , & Ferreira, C. (2021). How do ACT core processes underlie loneliness and psychological health? A study among people with and without physical chronic disease. Clinical Psychologist, 25(3), 329–338. 10.1080/13284207.2021.1979886

[bjc70072-bib-0007] Chou, T. L. , Chang, L. I. , & Chung, M. H. (2015). The mediating and moderating effects of sleep hygiene practice on anxiety and insomnia in hospital nurses. International Journal of Nursing Practice, 21(S2), 9–18. 10.1111/ijn.12164 26125570

[bjc70072-bib-0008] Chung, K. F. , Lee, C. T. , Yeung, W. F. , Chan, M. S. , Chung, E. W. Y. , & Lin, W. L. (2018). Sleep hygiene education as a treatment of insomnia: A systematic review and meta‐analysis. Family Practice, 35(4), 365–375. 10.1093/fampra/cmx122 29194467

[bjc70072-bib-0009] Daly‐Eichenhardt, A. , Scott, W. , Howard‐Jones, M. , Nicolaou, T. , & McCracken, L. M. (2016). Changes in sleep problems and psychological flexibility following interdisciplinary acceptance and commitment therapy for chronic pain: An observational cohort study. Frontiers in Psychology, 7, 1326. 10.3389/fpsyg.2016.01326 PMC5006108 27630601

[bjc70072-bib-0010] De Pasquale, C. , El Kazzi, M. , Sutherland, K. , Shriane, A. E. , Vincent, G. E. , Cistulli, P. A. , & Bin, Y. S. (2024). Sleep hygiene – What do we mean? A bibliographic review. Sleep Medicine Reviews, 75, 101930. 10.1016/j.smrv.2024.101930 38761649

[bjc70072-bib-0011] Dressle, R. J. , & Riemann, D. (2023). Hyperarousal in insomnia disorder: Current evidence and potential mechanisms. Journal of Sleep Research, 32(6), e13928. 10.1111/jsr.13928 37183177

[bjc70072-bib-0012] Faul, F. , Erdfelder, E. , Buchner, A. , & Lang, A.‐G. (2009). Statistical power analyses using GPower 3.1: Tests for correlation and regression analyses. Behavior Research Methods, 41(4), 1149–1160. 10.3758/BRM.41.4.1149 19897823

[bjc70072-bib-0013] Faul, F. , Erdfelder, E. , Lang, A.‐G. , & Buchner, A. (2007). GPower 3: A flexible statistical power analysis program for the social, behavioral, and biomedical sciences. Behavior Research Methods, 39(2), 175–191. 10.3758/BF03193146 17695343

[bjc70072-bib-0014] Friedrich, A. , & Schlarb, A. A. (2018). Let's talk about sleep: A systematic review of psychological interventions to improve sleep in college students. Journal of Sleep Research, 27(1), 4–22. 10.1111/jsr.12568 28618185

[bjc70072-bib-0015] Galvin, K. T. , Garland, S. N. , & Wibowo, E. (2022). The relationship between sleep hygiene, mood, and insomnia symptoms in men with prostate cancer. Support Care Cancer, 30(5), 4055–4064. 10.1007/s00520-021-06680-1 35064327

[bjc70072-bib-0016] Gilbert, K. E. , Tonge, N. A. , & Thompson, R. J. (2019). Associations between depression, anxious arousal and manifestations of psychological inflexibility. Journal of Behavior Therapy and Experimental Psychiatry, 62, 88–96. 10.1016/j.jbtep.2018.09.006 PMC6252139 30273794

[bjc70072-bib-0017] Gillanders, D. T. , Bolderston, H. , Bond, F. W. , Dempster, M. , Flaxman, P. E. , Campbell, L. , Kerr, S. , Tansey, L. , Noel, P. , Ferenbach, C. , Masley, S. , Roach, L. , Lloyd, J. , May, L. , Clarke, S. , & Remington, B. (2014). The development and initial validation of the cognitive fusion questionnaire. Behavior Therapy, 45(1), 83–101. 10.1016/j.beth.2013.09.001 24411117

[bjc70072-bib-0018] Grandner, M. A. , Olivier, K. , Gallagher, R. , Hale, L. , Barrett, M. , Branas, C. , Killgore, W. D. S. , Parthasarathy, S. , Gehrels, J.‐A. , & Alfonso‐Miller, P. (2020). Quantifying impact of real‐world barriers to sleep: The brief index of sleep control (BRISC). Sleep Health, 6(5), 587–593. 10.1016/j.sleh.2020.01.013 PMC12357651 32482573

[bjc70072-bib-0019] Harris, P. A. , Taylor, R. , Minor, B. L. , Elliott, V. , Fernandez, M. , O'Neal, L. , McLeod, L. , Delacqua, G. , Delacqua, F. , Kirby, J. , & Duda, S. N. (2019). The REDCap consortium: Building an international community of software platform partners. Journal of Biomedical Informatics, 95, 103208. 10.1016/j.jbi.2019.103208 PMC7254481 31078660

[bjc70072-bib-0020] Harris, P. A. , Taylor, R. , Thielke, R. , Payne, J. , Gonzalez, N. , & Conde, J. G. (2009). Research electronic data capture (REDCap)—A metadata‐driven methodology and workflow process for providing translational research informatics support. Journal of Biomedical Information, 42(2), 377–381. 10.1016/j.jbi.2008.08.010 PMC2700030 18929686

[bjc70072-bib-0021] Hayes, A. F. (2018). Introduction to mediation, moderation, and conditional process analysis: A regression‐based approach (2nd ed.). Guilford Press.

[bjc70072-bib-0022] Hayes, S. C. (2004). Acceptance and commitment therapy, relational frame theory and the third wave of behavioral and cognitive therapies. Behavior Therapy, 35, 639–665. 10.1016/S0005-7894(04)80013-3 27993338

[bjc70072-bib-0023] Hayes, S. C. , Luoma, J. B. , Bond, F. W. , Masuda, A. , & Lillis, J. (2006). Acceptance and commitment therapy: Model, processes and outcomes. Behaviour Research and Therapy, 44(1), 1–25. 10.1016/j.brat.2005.06.006 16300724

[bjc70072-bib-0024] Hertenstein, E. , Thiel, N. , Lüking, M. , Külz, A. K. , Schramm, E. , Baglioni, C. , Spiegelhalder, K. , Riemann, D. , & Nissen, C. (2014). Quality of life improvements after acceptance and commitment therapy in nonresponders to cognitive behavioral therapy for primary insomnia. Psychotherapy and Psychosomatics, 83(6), 371–373. 10.1159/000365173 25323449

[bjc70072-bib-0025] Kalmbach, D. A. , Buysse, D. J. , Cheng, P. , Roth, T. , Yang, A. , & Drake, C. L. (2020). Nocturnal cognitive arousal is associated with objective sleep disturbance and indicators of physiologic hyperarousal in good sleepers and individuals with insomnia disorder. Sleep Medicine, 71, 151–160. 10.1016/j.sleep.2019.11.1184 PMC8212183 32247571

[bjc70072-bib-0026] Lancee, J. , Eisma, M. C. , van Zanten, K. B. , & Topper, M. (2017). When thinking impairs sleep: Trait, daytime and nighttime repetitive thinking in insomnia. Behavioral Sleep Medicine, 15(1), 53–69. 10.1080/15402002.2015.1083022 26651373

[bjc70072-bib-0027] Looman, M. I. , Schoenmakers, T. M. , Blanken, T. F. , Linnebank, F. E. , JanH.Kamphuis, J. H. , & Lancee, J. (2024). Efficacy of acceptance and commitment therapy as a stand‐alone treatment for insomnia: Protocol of a randomized waitlist controlled trial. Journal of Behavioral and Cognitive Therapy, 34(3), 100499.

[bjc70072-bib-0028] Macri, J. A. , & Rogge, R. D. (2024). Examining domains of psychological flexibility and inflexibility as treatment mechanisms in acceptance and commitment therapy: A comprehensive systematic and meta‐analytic review. Clinical Psychology Review, 110, 102432. 10.1016/j.cpr.2024.102432 38615492

[bjc70072-bib-0029] Manzar, M. D. , Alghadir, A. H. , Khan, M. , Salahuddin, M. , Albougami, A. , Maniago, J. D. , Vasquez, B. A. , Pandi‐Perumal, S. R. , & Bahammam, A. S. (2021). Anxiety symptoms are associated with higher psychological stress, poor sleep, and inadequate sleep hygiene in collegiate young adults—A cross‐sectional study. Frontiers in Psychiatry, 12, 677136. 10.3389/fpsyt.2021.677136 PMC8280471 34276445

[bjc70072-bib-0030] Marques, D. R. , Gomes, A. A. , Ferreira, M. F. , & de Azevedo, M. H. P. (2016). Don't worry, sleep well: Predictors of sleep loss over worry. Sleep and Biological Rhythms, 14(3), 309–318. 10.1007/s41105-016-0060-z

[bjc70072-bib-0031] Mastin, D. F. , Bryson, J. , & Corwyn, R. (2006). Assessment of sleep hygiene using the sleep hygiene index. Journal of Behavioral Medicine, 29(3), 223–227. 10.1007/s10865-006-9047-6 16557353

[bjc70072-bib-0032] Mastin‐Purcell, L. , Richdale, A. L. , Lawson, L. P. , & Morris, E. M. J. (2025). Associations between psychological inflexibility processes, pre‐sleep arousal and sleep quality. Psychology and Psychotherapy: Theory, Research and Practice, 98, 606–623. 10.1111/papt.12584 PMC12346253 39976339

[bjc70072-bib-0033] McCracken, L. M. , Badinlou, F. , Buhrman, M. , & Brocki, K. C. (2020). The role of psychological flexibility in the context of COVID‐19: Associations with depression, anxiety, and insomnia. Journal of Contextual Behavioral Science, 19, 28–35. 10.1016/j.jcbs.2020.11.003

[bjc70072-bib-0034] McCracken, L. M. , Buhrman, M. , & Brocki, K. C. (2023). COVID‐19 and processes of adjustment in people with persistent pain: The role of psychological flexibility. Scandinavian Journal of Pain, 23(3), 539–545. 10.1515/sjpain-2022-0138 36779299

[bjc70072-bib-0050] Miniati, M. , Busia, S. , Conversano, C. , Orrù, G. , Ciacchini, R. , Cosentino, V. , Marazziti, D. , Gemignani, A. , & Palagini, L. (2023). Cognitive fusion, ruminative response style and depressive spectrum symptoms in a sample of university students. Life, 13(3), 803. 10.3390/life13030803 PMC10057694 36983958

[bjc70072-bib-0035] Morin, C. M. , Belleville, G. , Bélanger, L. , & Ivers, H. (2011). The insomnia severity index: Psychometric indicators to detect insomnia cases and evaluate treatment response. Sleep, 34(5), 601–608. 10.1111/j.1365-2648.2010.05394.x PMC3079939 21532953

[bjc70072-bib-0036] Palagini, L. , Hertenstein, E. , Riemann, D. , & Nissen, C. (2022). Sleep, insomnia and mental health. Journal of Sleep Research, 31(4), e13628. 10.1111/jsr.13628 35506356

[bjc70072-bib-0037] Paulos‐Guarnieri, L. , Linares, I. M. P. , & El Rafihi‐Ferreira, R. (2022). Evidence and characteristics of acceptance and commitment therapy (ACT)‐based interventions for insomnia: A systematic review of randomized and non‐randomized trials. Journal of Contextual Behavioral Science, 23, 1–14. 10.1016/j.jcbs.2021.11.001

[bjc70072-bib-0049] Peltz, J. (2024). Out of their control: College students’ beliefs in sleep myths, sleep problems, and psychological flexibility. Sleep Health, 10(4), 478–484. 10.1016/j.sleh.2024.05.002 38879397

[bjc70072-bib-0038] Peltz, J. S. , Rogge, R. D. , Bodenlos, J. S. , Kingery, J. N. , & Pigeon, W. R. (2020). Changes in psychological inflexibility as a potential mediator of longitudinal links between college students' sleep problems and depressive symptoms. Journal of Contextual Behavioral Science, 15, 110–122. 10.1016/j.jcbs.2019.12.003

[bjc70072-bib-0039] Ruan, J. , Chen, S. , Liang, J. , Mak, Y. W. , Yee Ho, F. Y. , Chung, K. F. , Kwun Tong, A. K. , Zhang, X. L. , & Yeung, W. F. (2022). Acceptance and commitment therapy for insomnia and sleep quality: A systematic review and meta‐analysis. Journal of Contextual Behavioral Science, 26, 139–155. 10.1016/j.jcbs.2022.09.002

[bjc70072-bib-0040] Ruan, J. Y. , Liu, Q. , Chung, K. F. , Ho, K. Y. , & Yeung, W. F. (2025). Effects of sleep hygiene education for insomnia: A systematic review and meta‐analysis. Sleep Medicine Reviews, 82, 102109. 10.1016/j.smrv.2025.102109 40449065

[bjc70072-bib-0041] Ryan, E. , O'Neill, D. , & Smyth, S. (2025). A systematic review and narrative synthesis of the complex interplay between psychological flexibility and sleep health. Journal of Contextual Behavioral Science, 35, 100871. 10.1016/j.jcbs.2025.100871

[bjc70072-bib-0042] Shin, J.‐W. , Kim, S. , Shin, Y. J. , Park, B. , & Park, S. (2023). Comparison of acceptance and commitment therapy (ACT) and cognitive behavior therapy (CBT) for chronic insomnia: A pilot randomized controlled trial. Nature and Science of Sleep, 15, 523–531. 10.2147/NSS.S409981 PMC10329838 37431325

[bjc70072-bib-0043] Van Someren, E. J. W. (2021). Brain mechanisms of insomnia: New perspectives on causes and consequences. Physiological Reviews, 101(3), 995–1046. 10.1152/physrev.00046.2019 32790576

[bjc70072-bib-0044] van Straten, A. , Weinreich, K. J. , Fábián, B. , Reesen, J. , Grigori, S. , Luik, A. I. , Harrer, M. , & Lancee, J. (2025). The prevalence of insomnia disorder in the general population: A meta‐analysis. Journal of Sleep Research, 34(5), e70089. 10.1111/jsr.70089 PMC12426706 40369835

[bjc70072-bib-0045] Vargas, I. , Nguyen, A. M. , Muench, A. , Bastien, C. H. , Ellis, J. G. , & Perlis, M. L. (2020). Acute and chronic insomnia: What has time and/or hyperarousal got to do with it? Brain Sciences, 10(2), 1–12. 10.3390/brainsci10020071 PMC7071368 32013124

[bjc70072-bib-0046] Wu, M. J. , Zhao, K. , & Fils‐Aime, F. (2022). Response rates of online surveys in published research: A meta‐analysis. Computers in Human Behavior Reports, 7, 100206.

[bjc70072-bib-0047] Zakiei, A. , Khazaie, H. , Rostampour, M. , Moradi, M. T. , Rezaie, L. , Komasi, S. , & Rafihi‐Ferreira, R. E. (2024). Associations between six core processes of psychological flexibility with poor sleep outcomes: A systematic review and meta‐analysis. Current Sleep Medicine Reports, 10(2), 257–275. 10.1007/s40675-024-00293-w

[bjc70072-bib-0048] Zhang, C. Q. , Zhang, R. , Lu, Y. , Liu, H. , Kong, S. , Baker, J. S. , & Zhang, H. (2021). Occupational stressors, mental health, and sleep difficulty among nurses during the COVID‐19 pandemic: The mediating roles of cognitive fusion and cognitive reappraisal. Journal of Contextual Behavioral Science, 19, 64–71. 10.1016/j.jcbs.2020.12.004 PMC7834481 33520643

